# Characterization of Phase Transition in the Thalamocortical System during Anesthesia-Induced Loss of Consciousness

**DOI:** 10.1371/journal.pone.0050580

**Published:** 2012-12-07

**Authors:** Eunjin Hwang, Seunghwan Kim, Kyungreem Han, Jee Hyun Choi

**Affiliations:** 1 Center for Neuroscience, Korea Institute of Science and Technology, Seoul, South Korea; 2 Asia Pacific Center for Theoretical Physics and Nonlinear Complex Systems Laboratory, Department of Physics, Pohang University of Science and Technology, Pohang, South Korea; 3 College of Pharmacy, Seoul National University, Seoul, South Korea; 4 Department of Neuroscience, University of Science and Technology, Daejon, South Korea; University of British Columbia, Canada

## Abstract

The thalamocortical system plays a key role in the breakdown or emergence of consciousness, providing bottom-up information delivery from sensory afferents and integrating top-down intracortical and thalamocortical reciprocal signaling. A fundamental and so far unanswered question for cognitive neuroscience remains whether the thalamocortical switch for consciousness works in a discontinuous manner or not. To unveil the nature of thalamocortical system phase transition in conjunction with consciousness transition, ketamine/xylazine was administered unobtrusively to ten mice under a forced working test with motion tracker, and field potentials in the sensory and motor-related cortex and thalamic nuclei were concomitantly collected. Sensory and motor-related thalamocortical networks were found to behave continuously at anesthesia induction and emergence, as evidenced by a sigmoidal response function with respect to anesthetic concentration. Hyperpolarizing and depolarizing susceptibility diverged, and a non-discrete change of transitional probability occurred at transitional regimes, which are hallmarks of continuous phase transition. The hyperpolarization curve as a function of anesthetic concentration demonstrated a hysteresis loop, with a significantly higher anesthetic level for transition to the down state compared to transition to the up state. Together, our findings concerning the nature of phase transition in the thalamocortical system during consciousness transition further elucidate the underlying basis for the ambiguous borderlines between conscious and unconscious brains. Moreover, our novel analysis method can be applied to systematic and quantitative handling of subjective concepts in cognitive neuroscience.

## Introduction

Many studies have characterized electrophysiological differences between conscious and unconscious states such as vegetative state and coma [1], anesthesia-induced unconsciousness [2]; however, the dynamic processes of electrophysiological activity during consciousness transitions have been poorly characterized. As a prototypical example, anesthesia is used to correlate altered neuronal function or cortical communication with loss of consciousness [3], [4]. Steyn-Ross *et al.* [5] formulated a theoretical model for the cerebral cortex response to general anesthesia and predicted a discontinuous phase transition of electrical fluctuations in the cortex at the point of transition into unconsciousness, which was characterized by a divergence in cortical synchrony and hysteresis in transitional paths. Their analogy of anesthetic phase transition to a thermodynamic phase change such as water freezing presumes that the anesthetic concentration is an external condition that drives the cortical state to an ordered phase, such as the unconscious state, by reducing neuronal excitability. Liley and Bojak [6] later adopted mean-field theory to correlate neuronal population behaviors with physiologically observable parameters (*e.g.*, electroencephalogram (EEG)), and Molaee-Ardekani *et al.* [7] added voltage-dependent slow motion of ions to the above models, predicting the existence of a broad regime of fluctuation between two states, which is a property of continuous phase transition. These models describe the mechanisms underlying why and how anesthesia drives cortical neurons from the up state (depolarized mode) to the down state (hyperpolarized mode). However, there is no quantifiable variable representing the onset of consciousness transition suggested in freely behaving individual animal, as far as we know.

It has been shown that the resonance oscillations with large amplitude in the thalamocortical network are correlated with the functional states of cortical neurons [8], [9]. A synchronous alteration of thalamocortical neuron up (depolarized) and down (hyperpolarized) states blocks afferent information and disassociates top-down integration. In the present study, the generation and breakdown of resonant oscillations in the thalamocortical network associated with anesthesia were investigated in sensory and motor thalamocortical circuit, with a novel analysis method quantifying an order parameter which is 0 and 1 in information transmission and blocking modes, respectively. Our primary purpose was to characterize thalamocortical network phase transition in the induction and emergence of anesthesia, with the aim of locating the critical points of transition to unconsciousness or consciousness in the thalamocortical network. Consciousness is a subjective term, and our strategy is to delimit the unconsciousness as a loss of ability to integrate perceptual and motor events together with memory to respond adequately to a given situation, which is referred to primary consciousness by Edelman [10]. The moments of behavioral change induced by anesthetic administration was pinpointed with motion tracker and three-compartment modeling was employed to estimate the ketamine concentration in the brain. The dynamic behavior of the sensorimotor thalamocortical network was characterized according to external conditions such as drug induction time and anesthetic concentration in the brain.

## Materials and Methods

### Surgical Procedures

The mouse model was used in research and all surgical and experimental procedures were conducted in accordance with the guidelines for the Institutional Animal Care and Use Committee, following Act 1992 of the Korea Lab Animal Care Regulations and associated guidelines.

**Figure 1:**
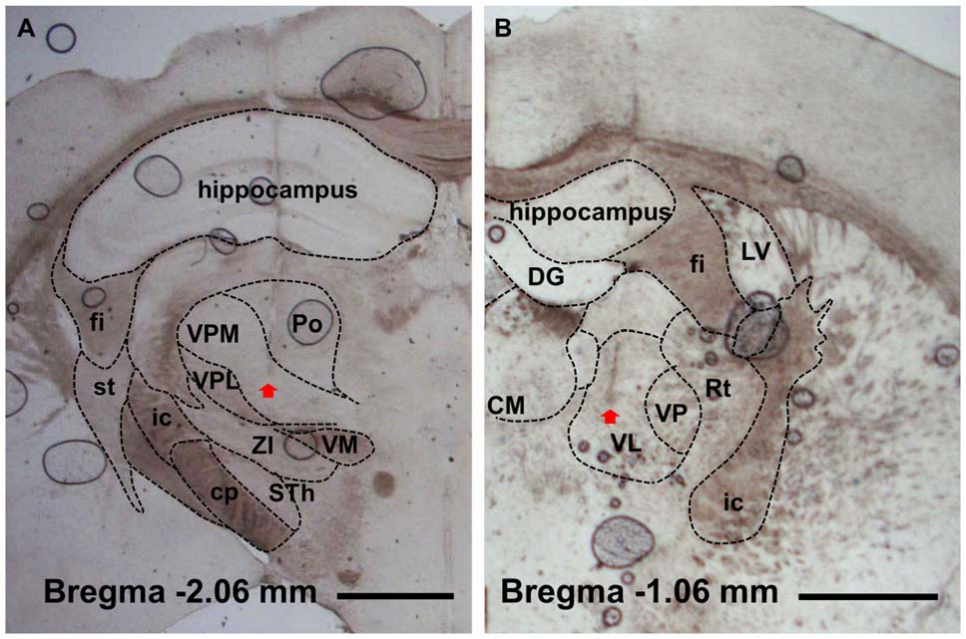
Verification of electrodes location. Electrode placements in ventral posteromedial (A) and ventral lateral (B) nuclei were histologically verified with light microscopy. Arrow point indicates the tip of electrode. fi, fimbria of the hippocampus; st, stria terminalis; ic, internal capsule; cp, cerebral peduncle (basal part); VPM, ventral posteromedial nucleus; VPL, central posterolateral nucleus; ZI, zona incerta; STh, subthalamic nucleus; Po, posterior thalamic nuclear group; VM, ventromedial thalamic nucleus; DG, dentate gyrus; CM, central medial nucleus; VL, ventral lateral nucleus; VP, ventral posteromedial and posterolateral nuclei; Rt, reticular thalamic nucleus; LV, lateral ventricle. Scale bar is 1 mm.

Experiments were conducted on ten male C57BL/6×129 F1 hybrid mice (8–10 weeks; body weight 19–25 g). Surgical procedures were performed as described in a previous report [11]. In brief, mice under ketamine-xylazine anesthesia (120 and 6 mg/kg, respectively, i.p.) were implanted with two tungsten electrodes (Teflon-insulated tungsten wire 76.2/114.3 µm in bare/coated diameter, A-M Systems, Sequim, WA, USA) positioned at the ventral lateral (1.06 mm posterior, 1.1 mm lateral and 3.5 mm ventral to bregma) and ventral posteromedial nuclei (1.82 mm posterior, 1.5 mm lateral and 3.7 mm ventral to bregma) of the thalamus; two cortical screw electrodes (chrome-plated stainless steel 3 mm in length and 1 mm in diameter, Asia Bolt, Seoul, Korea) positioned at the primary motor (0.74 mm anterior, 1.5 mm lateral and 0 mm ventral to bregma) and primary somatosensory cortices (1.82 mm posterior, 3.0 mm lateral and 0 mm ventral to the bregma); and a ground electrode on the interparietal bone. All electrode coordinates followed the mouse brain atlas [12]. After experiments, histology was performed to verify the thalamic placement (Fig. 1), and all the data with tip of electrode located out of the targeted thalamic nuclei were excluded in the analysis. For remote administration of the anesthetic agent during electrophysiological recording, polyethylene tubing (10 cm in length, 1520/86 µm in outer/inner diameter; PE 100, Intramedic polyethylene tubing, Clay Adams, Sparks, MD, USA) was inserted in the intraperitoneal cavity and sutured.

### Electrophysiological Recordings under Forced Walking

Electrophysiological recording was performed under a forced walking scheme to prevent EEG artifacts induced by the experimenter’s intervention during anesthetic drug injection and to obtain behavioral reference points of brain state transitions [11]. Prior to experiments, each mouse was habituated to a treadmill (LE8708, Panlab, Spain) in a suspended condition for approximately 15 min and in a walking condition for approximately 5 min. While the animal was walking on the treadmill, baseline EEG signals were recorded for 10 min, followed by unobtrusive administration of the ketamine–xylazine cocktail (120 and 6 mg/kg, respectively) through the preinstalled injection tube. Recording continued until the animal awakened and recovered its movement. The motion of the animal was simultaneously monitored by an accelerometer-based motion sensor (MMA7260Q, Freescale Semiconductor Inc., Austin, TX, USA) attached to the animal’s head and used to detect behavioral reference points of loss and recovery of motion referred to as *t*_LOM_ and *t*_ROM_, respectively, with respect to the time of drug administration (*t*_ADM_). The speed of the lane was maintained at 5 cm/s throughout the recording period, which induced minimal walking behavior for the animal equivalent to a spontaneously behaving condition. The sides of treadmill were surrounded with a wall to keep the animals on the treadmill. After loss of motion, the animals lied down above the running lane against the wall continuously receiving tactile stimulus generated by friction from the lane surface.

Simultaneously with motion signal acquisition, EEGs were acquired in a monopolar scheme referenced to the ground electrode by an analog amplifier (8–16C, Grass Technologies, West Warwick, RI, USA) and digitized with an analog-digital converter (Digidata 1440A, Molecular Devices, Sunnyvale, CA, USA) at a sampling frequency of 10 kHz. Before analysis, EEG signals were band-pass filtered with cut-off frequencies of 0.5 and 100 Hz (linear-phase Bessel filter), and then the sampling frequency was reduced to 200 Hz by moving average. Each signal was normalized by its average power in the frequency range of 90–100 Hz during the quiescent period.

### Phase Locking Value as an Index of Phase Synchronization in Delta Band

Phase synchronization was calculated with phase locking value (PLV) defined as
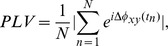
where 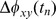 is the phase difference between signal *x* and *y* at time *t_n_* and N is the number of data points in a 10-s analysis window. 1–4 Hz band-pass filtered EEG signals (zero-phase filtering with FIR filter of order 400) were Hilbert transformed to yield instantaneous phases and the PLVs between motor-related thalamocortical pair and somatosensory-related pair were computed.

### Dimension Reduction with Aggregate Time Units

To reduce the complexity of the EEG signals, we introduced aggregate time epochs with variable intervals reflecting the period of one slow fluctuation. The aggregate time was defined as the point where more than three neuronal signals got into negative peaks simultaneously. The negative peaks of the signals were designated as the points satisfying the criterion that the Hilbert phase of the 1–4 Hz band-pass filtered signal (zero-phase filtering with FIR filter of order 400) equaled –π with 15% tolerance.

### Concept of Order Parameter and Quantification of Information Blocking

When we assume each region represents a functional unit in terms of neuronal information transfer, the oscillation patterns in each epoch reflect functional states of the brain. To each state of a brain system functional unit, there corresponds a “state vector” in the description of its resonant oscillation. For example, suppose that a functional brain unit is perfectly resonant within the thalamocortical system, rarely responding to any perturbation and resultantly blocking signal transmission. We can designate the corresponding state vector as 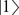 when all the functional units in the brain system are synchronously in a resonant state, whereas 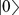 when none of the units experiences resonant oscillation.

The notation 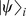 is used for the state of the thalamocortical system at the *i^th^* epoch to represent the brain state as
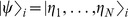
(1)for *N* different functional units, *η_j_*. An operator, *A*, acting on the state vector to evaluate brain state can be written as a superposition of independent operator *A_j_* acting on the state vector of each functional unit, *i.e.*,



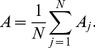
(2)For each operator, a state value for the *j^th^* functional unit at the *i^th^* epoch can be determined as 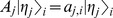, where *a_j,i_* is 1 or 0 for ordered and disordered states, respectively. Here, an order parameter of interest, 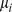 can be defined as
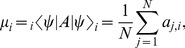
(3)with the value of 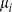 ranging from 0 to 1. The signal operator was defined as
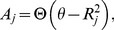
(4)where 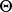 is a Heaviside step function, θ is a predetermined order-disorder threshold, and 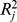 is a dissimilarity function. Dissimilarity was calculated by comparing the length- and amplitude-normalized oscillations in each epoch with a template of a representative epoch from deep anesthesia, which was drawn by normalizing and averaging up to 500 epochs sampled from 10 min after the drug administration. Dissimilarity function for the i^th^ epoch, 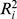, was calculated as
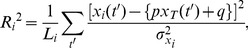
(5)where 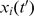 is the signal of the ith epoch in normalized time t′, 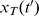 is the template signal in normalized time t′, 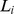 is the length of 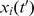, 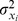 is the variance of 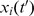, and p and q are least-square fitting parameters minimizing 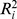. In the present study, θ was determined by k-means clustering of a sampled 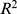 distribution from wakeful and anesthetized periods (k = 2). 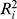 value is conceptually similar to a z-score where larger absolute z-scores indicate larger deviation from the mean value. A pattern of the i^th^ epoch is similar to the normalized template when 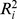 is close to zero, and it is more deviant from the template as 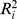 has larger value.

To estimate the readiness for transition between wakeful state and anesthesia, the susceptibility of the order parameter to the anesthetic concentration during anesthetic induction and recovery was calculated as 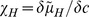 and 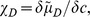 respectively, where 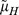 and 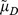 are sigmoidal fitting functions of *μ* during anesthetic induction and recovery, respectively, and *c* is the anesthetic concentration estimated from mathematical modeling (see below section for mathematical modeling of anesthetic concentration). Anesthetic induction was defined as the period in which the estimated anesthetic concentration was in the increasing phase before reaching its maximum, and recovery as the period during which the anesthetic concentration decreased after the maximum. The shape of the sigmoidal fitting function followed 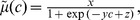 and the fitting coefficients *x*, *y*, and *z* were estimated in a least squares manner. During the anesthetic induction period, the more easily hyperpolarized neuron groups were expected to have a higher level of susceptibility. Hence, susceptibility during induction was referred to as the “hyperpolarizing susceptibility,” 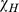. By contrast, susceptibility during recovery gauges the ability to resume information transfer by breaking the resonant oscillations; therefore, it was referred to as the “depolarizing susceptibility,” 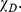


### Probability of Transition between States

The probability of transition between states, *d*, in a 10-s observation window was defined as
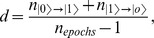
where 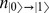 and 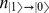 are the number of state transitions switching from a disordered (up state) to an ordered state (down state) and vice versa, and 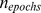 is the total number of epochs in the observation period, with an epoch defined by the time interval between adjunct aggregate time points. A zero value of *d* indicates that the thalamocortical system stays in a single state during the observation period, and positive unity indicates that the thalamocortical system continuously switches its state.

### Mathematical Modeling of Anesthetic Concentration in the Brain

Prior to further investigating the characteristics of state transition, determining whether time is the appropriate variable to describe the brain state is a crucial question. Assuming susceptibility to anesthesia depends on the concentration of the anesthetic in the cerebral circulatory system [13], the concentration of ketamine in the cerebral circulatory system *was considered the* reference parameter reflecting the endogenous state of the brain. However, directly measuring this concentration is difficult in practice due to the limited access to the dense network of blood vessels in the brain in the electrode-implanted mice. Indirect measurement of ketamine concentration through the peripheral plasma also has limitations as it inevitably affects EEG recording, since the needle’s sting may distort EEG signals or induce EEG artifacts during blood sampling. To circumvent these limitations, a mathematical model based on the behavioral reference points, *i.e.*, *t*_ADM_, *t*_LOM_, and *t*_ROM_, which are believed to be directly proportional to the cerebral levels of ketamine, was used to predict the ketamine concentration in the brain.

The mathematical model consists of three compartments. The first (C_1_) includes the initial dilution volume, which is composed of the rapidly perfused tissues and organs such as the brain. The second compartment (C_2_) involves the tissues and organs of the body that are less well perfused. Finally, an additional compartment (C_a_) that denotes the site of ketamine administration is incorporated into the model. The dynamics equations for the time evolution of the ketamine concentrations in the C_a_, C_1_, and C_2_ compartments, designated by *c*_a_, *c*_1_, and *c*_2_, respectively, are determined by the difference between corresponding influx and efflux rates. These are expressed in terms of various specific rates, which describe elimination of ketamine (*k*_a_) and transitions of ketamine (*k*_12_ and *k*_21_). The details of the model system are described in Information S1 with the model parameters in Table S1.

The resulting equation for the ketamine concentration in C_1_ after administration is given by:
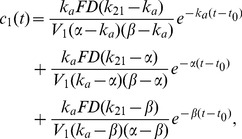
(6)where *k*_a_ is the rate constant for drug absorption from the administration compartment, *k*_21_ is the rate constant for drug transition from C_1_ to C_2_, *α* is the rate constant for ketamine distribution process, *β* is the rate constant for ketamine elimination, *F* is the bioavailability, *D* is the loading dose, *V*_1_ is the volume of C_1_, and *t*_0_ is thedrug administration time. *k*_a_ and *α* were determined to be inverse-proportional to the intervals between *t_ADM_* and *t_LOM_*, and between *t_LOM_* and *t_ROM_*, respectively, which were considered as proxies for anesthetic effects. The estimated ketamine concentration *c*_1_ was normalized by its maximum value to yield values between 0 and 1. Later the normalized anesthetic concentration was denoted as *c* without a subscript.

## Results

### Delayed Generation and Advanced Breakdown of Thalamocortical Slow Oscillation with Respect to Loss and Recovery of Motion

**Figure 2:**
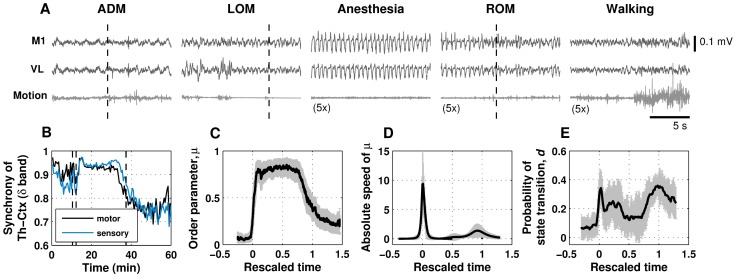
Dynamics of brain state in temporal domain. A. Representative time series for an electroencephalogram (EEG) from primary motor cortex (M1), local field potentials from ventral lateral (VL) thalamus, and motion signal around the time of drug administration (ADM), loss of motion (LOM), deep anesthesia, recovery of motion (ROM), and resumption of walking. For the periods of anesthesia, recovery of motion and resumption of walking, the motion signal is exaggerated by five times for clearance. Dashed lines in the panels indicate corresponding events. B. Representative plot of δ band (1–4 Hz) phase synchronization for thalamocortical motor pair (M1 and VL) and sensory pair (primary somatosensory cortex and ventral posteromedial nucleus). Dashed lines indicate drug administration (*t_ADM_*), loss of motion (*t_LOM_*), and recovery of motion (*t_ROM_*), from left to right. C. Ensemble average (N = 10) of the order parameter (black solid) and its standard error mean (gray errorbars) as a function of rescaled time. The rescaled time is obtained by normalizing measurement time with respect to the blackout period. The moments of *t_LOM_*, and *t_ROM_* correspond to 0 and 1, respectively. D. Ensemble-averaged absolute value of time derivative of order parameter (black solid) and its standard error mean (gray errorbars). The peak amplitude during anesthetic induction period was statistically significantly larger than the value at recovery (t-test, *P<*0.05). E. Probability of transition between up and down states at 10-s time intervals. Gray errorbars indicate standard error mean of the transition probability. For a given 10-s observational period, the number of epochs was approximately twenty during the transitional periods and fifteen during the deep anesthesia period.

In Fig. 2A, 15-s sample signals of EEG and motion show qualitative differences of brain states in the vicinity of *t_ADM_*, *t_LOM_*, deep anesthesia, *t_ROM_*, and resumption of walking. The EEG signals in the middle of anesthesia period have regularly-repeating large amplitude oscillations in delta band range (1–4 Hz). In delta band, phase synchronization values for the motor-related thalamus-cortex pair and the somatosensory-related thalamus-cortex pair were increased and kept high during the anesthetic period (Fig. 2B).

The change of electrophysiological brain state and its saturation did not synchronously match the change in behavior response under anesthesia: the change of the brain state lagged behind *t_LOM_* during induction of anesthesia, and this temporal relationship was reversed during emergence, *i.e.*, by advancing *t_ROM_*. Figure 2C shows the subject-averaged order parameter *μ* over rescaled time, which is normalized against the blackout time (*i.e.*, period between *t_LOM_* and *t_ROM_*). The induction time (*i.e.*, period between *t_ADM_* and *t_LOM_*) was 96.7±36.8 s (mean±SD) and the blackout time was 33.2±8.3 min (mean±SD). As indicated by the response curve (Fig. 2C), a time delay occurred before the order parameter reached its saturation value. Although the saturation points were roughly estimated due to the fluctuation in their vicinity, our estimation of the time delay of the saturation point with respect to *t_LOM_* was 2.0±0.6 min, and the time advance of the saturation point with respect to *t_ROM_* was 8.6±4.8 min. The average duration of the steady state of the order parameter was 22.7±5.3 min.

### Quick Induction and Slow Emergence of Anesthesia in the Time Domain

The brain state transition to the down state during induction of anesthesia was noted to occur significantly faster than the transition to the up state during emergence from anesthesia. The difference in the speed of transition is reflected by the absolute values of the time derivative *μ* in Fig. 2D, which describes the speed of phase transition. The generation of global synchronization to resonant oscillations was found to occur responsively within a few minutes in the induction period, whereas the disassociation of the resonant oscillations in the emergence period proceeded slowly toward the recovery of motion over a time course of several minutes.

### Fluctuations between a Hyperpolarized Down State and Depolarized up State

Figure 2E shows the transitional probability between up and down states, *d*, within a 10-s interval. Notably, the time of maximal transitional probability coincided with the time of maximal transitional speed during the induction period in Fig. 2D. By contrast, the transitional probability began to increase at the end of the steady state of *μ* until *t_ROM_* and did not drop to zero even after *t_ROM_*. The large temporal fluctuation between up and down states implies that the direction of transition during the transitional period is not fixed, strongly suggesting the non-deterministic nature of consciousness transition around transitional period.

### Path-dependent Reaction to Anesthesia Induction and Washout

**Figure 3:**
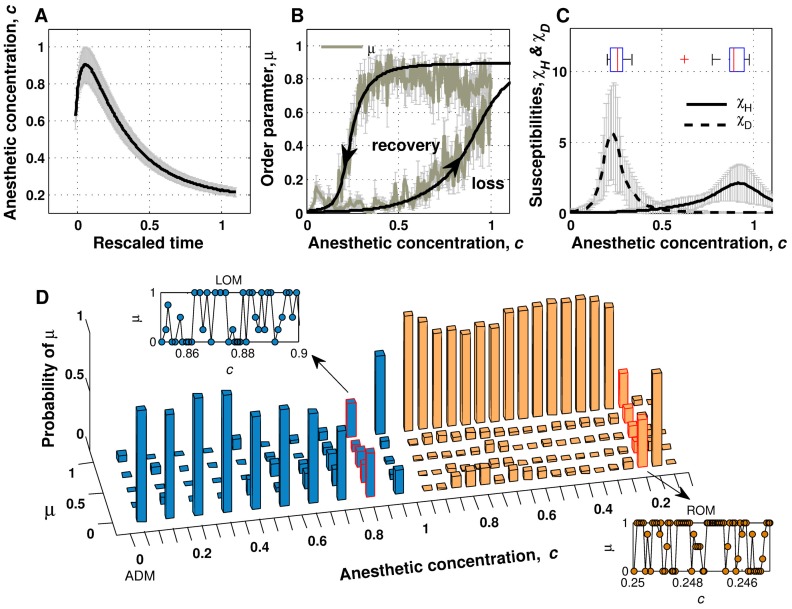
Path-dependent change of brain state and metastable fluctuation during transition. A. Anesthetic concentration estimated with the three-compartment is plotted with respect to rescaled time, where the moments of *t_LOM_*, and *t_ROM_* correspond to 0 and 1, respectively. Black solid line indicates averaged anesthetic concentration among ten subjects and gray errorbars indicate standard error mean. B. Ensemble average (N = 10) of the order parameter (greenish gray solid) and sigmoidal curve fitting as a function of anesthetic concentration *c* (black solid). Gray errorbars indicate standard error mean of the order parameter. Arrowheads show the passage of time. C. Ensemble-averaged time courses of hyperpolarizing (solid) and depolarizing (dashed) susceptibilities. Gray errorbars indicate standard error mean of the susceptibilities. The distributions of concentration for maximal susceptibility are shown as the embedded boxplots at the corresponding peaks. D. Representative plot of the recurrence probability of the order parameter for each bin of anesthetic concentration (bin size = 0.1/0.05 for the induction and emergence periods, respectively). Traces of the order parameter at the transitional moments at approximately 0.8 (during induction of anesthesia) and 0.2 (during recovery) are provided as inset figures. The anesthetic concentration of 0.0 at the left side of the axis indicates the moment of drug administration (ADM).

As previously stated, we observed large individual variation in drug induction time and blackout time across ten animals. We assumed the drug induction and blackout times as the time constants for drug absorption and early wash-out, respectively, and estimated the ketamine concentration in the brain using a three-compartment model (Fig. 3A). The subject-averaged response functions of *μ* as a function of anesthetic concentration *c* are plotted in Fig. 3B with curve fitting. The lag of *μ* is observed in both transitional paths, showing a hysteresis loop. This observed path-dependent nature of brain systems during transitions in consciousness is consistent with theoretical predictions [5] and path-dependent behavioral responses to anesthetic dose [14]. Furthermore, it is interesting to note that the hyperpolarizing susceptibility, 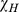
(*i.e.*, Δ*μ/*Δ*c* in the induction period) reached its maximum at the anesthetic concentration value of 0.875±0.106, which is significantly higher than the critical value of 0.257±0.044 for the depolarizing susceptibility, 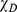 (*i.e.*, Δ*μ/*Δ*c* in the emergence period) as shown in Fig. 3C. This result implies that a certain level of anesthetic concentration is needed for global synchronization, but neuronal recruitment grows extensively near the point of highest anesthetic concentration. The lower threshold for depolarization compared to the threshold for hyperpolarization suggests that once neurons are hyperpolarized, they have a tendency to maintain their resonant oscillations.

### Temporary Metastable State of Global Activity during Transitional Periods

The calculated probability distribution of *μ* as anesthetic concentration changes is shown in Fig. 3D. Interestingly, mixed states exist near transitional points with fractional values of *μ*, *e.g.*, near 0.8 in the increasing phase of *c* and near 0.2 in the decreasing phase of *c*. These bimodal peaks observed at the transitional periods imply the existence of spatially and temporally local up or down states in the thalamocortical network.

A close look at the microscopic behaviors of *μ* suggests a co-existence of two states during the transitional periods: Representative figures of the temporal dynamics of *μ* in both periods are presented with respect to the change of anesthetic concentration in the inset of Fig. 3D. Although the averaged behavior of the order parameter favors state 0 or 1 statistically, prolonged microscopic spatial fluctuations exist, which is a characteristics of meta-stability. The temporal fluctuations of up and down states in each location of the thalamocortical network are visualized in Movie S1. Taken together, these results indicate that the thalamocortical system never shuts down instantaneously in ketamine-induced loss of consciousness. Moreover, the existence of meta-stability implies that blockade and recovery of information transfer within the thalamocortical network are initiated at discrete regions fluctuating over a wide range of anesthetic concentrations, which eventually drives the whole system into one phase in a hysteretic manner.

## Discussion

We defined the order parameter from experimentally obtained neuronal signals to determine the level of information blockade within the thalamocortical circuit by adopting aggregate time units and resultantly reducing the complexity of the field potential signals. Application of the aggregate time unit made it possible to split the time series into epochs containing electrophysiologically meaningful oscillations. Depending on whether each epoch showed thalamocortical resonant oscillation, we were able to determine the state vector and order parameter. Although a similar description of the system can be achieved by conventional indices such as the spectral edge frequency 95^th^ percentile [15], [16], the discretization method established here has the advantage of tracing changes of state statistically over a biologically relevant time scale. By distinguishing each discrete epoch into an oscillatory pattern of unconsciousness or not, fluctuation between states can be discerned, and the probability of transition between states can be defined.

The probability of transition between states was observed to increase in a time-locked manner to the loss and recovery of motion, indicating increased fluctuation around the behavioral transition points, and this prolonged spatial and temporal fluctuation between states implies that the transitions may not be discrete at the whole brain level. Although the anesthetic-induced transition model of Steyn-Ross et al. [5] proposed that a single macrocolumn in cerebral cortex may undergo first-order state transition with hysteresis, they also reported that the more realistic cortical model of 2-D grid of macrocolumns did not simultaneously go off to the unconscious state due to vagarious subcortical driving force [17]. Later modeling studies showed that the anesthesia-induced state transition could also occur in a non-discrete way through Hopf bifurcation, by expanding the model to include anesthetic effects on both duration and amplitude of IPSP [6] and slow ionic currents [7]. The response curve of order parameter with respect to anesthetic concentration captured not only the path-dependent nature that the local cerebral cortical unit might have, but also the system level fluctuations during state transitions.

The investigation of the order parameter in the temporal domain revealed lag and lead between behavioral representation and the change in thalamocortical information blocking level. However, the blackout time from *t_LOM_* to *t_ROM_* demonstrated large individual variance which was mainly attributed to the variance of *t_ROM_*, raising the question that whether time is the most appropriate reference for phase transition in consciousness. Since investigation in the temporal domain might not be sufficient to determine whether the difference in the order parameter rate of change originated from pharmacokinetic or neural mechanisms, we introduced a mathematical model to infer anesthetic concentration in the brain tissue.

The anesthetic concentration model was built as a three-compartment model considering the intraperitoneal injection route, where the model parameters were determined from previous pharmacokinetic studies. Basically the model parameters were estimated from the ketamine data, and the alteration of the ketamine’s anesthetic effect that co-administration of xylazine could give rise was implicitly incorporated in the model parameters *k_a_* and *α*, which were determined to be proportional to induction time and blackout time, respectively. While the pharmacokinetics data of xylazine for mice was rarely available, co-administration of xylazine was reported to affect the action of other anesthetics in such ways of lengthening the anesthesia period in budgerigars [18] or reducing the amount of anesthetic drug used for surgical anesthesia in cats [19]. The behavioral transition points were thought as the alternative indicators of overall anesthetic effects including probably delayed recovery from ketamine anesthesia due to xylazine.

Although we adopted the behavioral transition points as proxies of anesthetic effects, the absence of simultaneously measured plasma concentration is a manifest limitation that cannot be easily evaded in estimating the anesthetic concentration. To verify the robustness of the model, its dependence on the parameter selection was tested with varying parameters of *k*_a_ and *α* which were crucial for shaping the concentration model. As intraperitoneally injected drug is not absorbed into the circulation system directly, estimated *k*_a_, a rate constant for drug absorption from the intraperitoneal cavity, can have some variance depending on each subject’s condition. While the other parameters were fixed, variation of *k*_a_ up to ±50% of the estimated parameter did not make qualitative changes in the order parameter curve with regard to anesthetic concentration (Fig. S2(a) in Information S2). Variation of *α*, which might be raised by xylazine, resulted in wider range of variability in the order parameter curve, but the path-dependent nature was conserved even with ±50% variation of *α* value (Fig. S2(b), Information S2). The response function of thalamocortical states with respect to anesthetic concentration demonstrated significantly lower individual variance than that with respect to time, as well as maintained its hysteretic feature regardless of variable concentration model parameters, suggesting that the anesthetic concentration is a better reference for predicting the thalamocortical system state.

The observation that a higher anesthetic concentration was required for loss of consciousness compared to the recovery period provides experimental evidence for the tendency of the brain system to resist any change in state, and supports the idea that neural activities experience neural inertia. The term “neural inertia” was originally introduced by Friedman et al. to describe the path-dependent behavioral responses to anesthetic dose [14]. In their work, scatter plots of behavioral responses versus anesthetic doses measured in a population demonstrated hysteresis, and Hill slopes were steeper during the induction of anesthesia compared to emergence. The smaller time derivative values of the order parameter during recovery in our study are consistent with the interpretation that the reconstitution of consciousness is more difficult than shutting it down. Our results add electrophysiological evidence that a barrier exists between the wakeful state and anesthesia-induced unconscious state even in an individual brain.

Here we have assumed that the resonant oscillation in the thalamocortical network reflects the level of information blocking, and thus the level of vigilance. The thalamocortical pathway is thought to play an important role in sensory information transfer, raising the concept of thalamic consciousness switch [20]. Corticocortical integration has also been postulated as essential for consciousness [4], [21], with the experimental evidence that the corticocortical information integration level has been observed to significantly decrease during sleep and anesthesia [3], [22]. Although the extent to which these mechanisms contribute for consciousness may be argued, it seems that these two mechanisms are not exclusive but rather constitutes a hierarchical structure for the emergence of consciousness. The mismatch between the blackout period and the steady state of order parameter might originate from sequential recovery of this hierarchy. We cannot say that the order parameter which quantifies the level of thalamic information blocking as the only and all-powerful index for measuring consciousness level, but we can say that the order parameter reflects the change of the primary level in the hierarchical mechanism for consciousness.

From the order parameter diagram represented as a function of anesthetic concentration, we observed the existence of meta-stability and path-dependency in the periods of consciousness transition induced by anesthesia. In our study, it is conceptually interpreted as the expectation value of the global resonance level among different functional units. Not restricted to this single approach, the order operator can be defined in various ways depending on the target system’s characteristics and utilized independently of measurement modality and number of measurement sites. The formulation described in this work can promote the quantitative investigation of brain state transition characteristics. Unquestionably, ketamine/xylazine anesthesia is not the single route to unconsciousness, and the resonant oscillations of the thalamocortical system are not the unitary correlate of unconsciousness; however, this bottom-up approach has the advantage of enabling a gradual understanding of the “states” of consciousness.

## Supporting Information

Figure S1 **Diagram of three-compartment model. Xi denotes the amount of ketamine in the compartment Ci.** The transition rates between compartments (kij) and the elimination rates from the site of drug administration (ka) and from the cerebral circulatory system (k10) are assumed to be first order. D and F mean dose of ketamine and bioavailability, respectively. The ketamine concentration at Ci is defined as the amount of ketamine at the compartment divided by the volume of the compartment.
(TIF)

Figure S2 **(a) Variation of order parameter curve with respect to varying** ***k_a_*** **.** Order parameter curves are drawn with the anesthetic concentration profiles calculated with 50, 70, 90, 110, 130 and 150% values of estimated *k_a_*, from left to right. (b) Variation of order parameter curve with respect to varying α. Order parameter curves are drawn with the anesthetic concentration profiles calculated with 50, 70, 90, 110, 130 and 150% values of estimated α, from left to right. (c) Variation of order parameter curve with respect to varying *k_12_*. Order parameter curves are drawn with the anesthetic concentration profiles calculated with 50, 70, 90, 110, 130 and 150% values of estimated *k_12_*, from left to right. (d) Variation of order parameter curve with respect to varying β. Order parameter curves are drawn with the anesthetic concentration profiles calculated with 50, 70, 90, 110, 130 and 150% values of estimated β, from left to right.
(TIF)

Table S1 **The parameters without asterisks are fixed ones, determined from the plasma concentration-time profiles of ketamine.** Those with asterisks are adjustable ones, derived from the time profile of plasma levels of ketamine and/or the behavioral reference points i.e., *t*_ADM_, *t*_LOM_, and *t*_ROM_. The values with dagger (^†^) were expressed as means±standard deviation.
(DOCX)

Movie S1 **Spatio-temporal fluctuation of states during transition periods.** Upper panel shows fluctuation of the order parameter computed at the aggregate time (T) from each recording site (clockwise from top left: primary motor cortex, primary somatosensory cortex, somatosensory thalamus and motor thalamus) in the vicinity of *t_ADM_*, *t_LOM_* and *t_ROM_*. White and black colors indicate information transmission mode and information blocking mode, respectively. In the order parameter diagram at lower panel, moving red bar shows a position of corresponding aggregate time. Dashed vertical lines indicate *t_ADM_*, *t_LOM_* and *t_ROM_*, from left to right.
(MP4)

Information S1 (DOCX)

Information S2 (DOCX)

## References

[1] Laureys S, Owen AM, Schiff ND (2004) Brain function in coma, vegetative state, and related disorders. Lancet Neurol 3: 537–546. 15324722 10.1016/S1474-4422(04)00852-X

[2] Boveroux P, Vanhaudenhuyse A, Bruno MA, Noirhomme Q, Lauwick S, et al (2010) Breakdown of within- and between-network resting state functional magnetic resonance imaging connectivity during propofol-induced loss of consciousness. Anesthesiology 113: 1038–1053. 20885292 10.1097/ALN.0b013e3181f697f5

[3] Ferrarelli F, Massimini M, Sarasso S, Casali A, Riedner BA, et al (2010) Breakdown in cortical effective connectivity during midazolam-induced loss of consciousness. Proc Natl Acad Sci U S A 107: 2681–2686. 20133802 10.1073/pnas.0913008107 PMC2823915

[4] Alkire MT, Hudetz AG, Tononi G (2008) Consciousness and anesthesia. Science 322: 876–880. 18988836 10.1126/science.1149213 PMC2743249

[5] Steyn-Ross ML, Steyn-Ross DA, Sleigh JW (2004) Modelling general anaesthesia as a first-order phase transition in the cortex. Prog Biophys Mol Biol 85: 369–385. 15142753 10.1016/j.pbiomolbio.2004.02.001

[6] Liley DT, Bojak I (2005) Understanding the transition to seizure by modeling the epileptiform activity of general anesthetic agents. J Clin Neurophysiol 22: 300–313. 16357635

[7] Molaee-Ardekani B, Senhadji L, Shamsollahi MB, Vosoughi-Vahdat B, Wodey E (2007) Brain activity modeling in general anesthesia: enhancing local mean-field models using a slow adaptive firing rate. Phys Rev E Stat Nonlin Soft Matter Phys 76: 041911. 17995030 10.1103/PhysRevE.76.041911 PMC2117372

[8] Steriade M, McCormick DA, Sejnowski TJ (1993) Thalamocortical oscillations in the sleeping and aroused brain. Science 262: 679–685. 8235588 10.1126/science.8235588

[9] Llinas RR, Steriade M (2006) Bursting of thalamic neurons and states of vigilance. J Neurophysiol 95: 3297–3308. 16554502 10.1152/jn.00166.2006

[10] Edelman GM (2003) Naturalizing consciousness: a theoretical framework. Proc Natl Acad Sci U S A 100: 5520–5524. 12702758 10.1073/pnas.0931349100 PMC154377

[11] Hwang E, Kim S, Shin HS, Choi JH (2010) The forced walking test: a novel test for pinpointing the anesthetic-induced transition in consciousness in mouse. J Neurosci Methods 188: 14–23. 20117136 10.1016/j.jneumeth.2010.01.028

[12] Paxinos G, Franklin KBJ (2001) The Mouse Brain in Stereotaxic Coordinates. San Diego, CA: Academic Press.

[13] Schuttler J, Stanski DR, White PF, Trevor AJ, Horai Y, et al (1987) Pharmacodynamic modeling of the EEG effects of ketamine and its enantiomers in man. J Pharmacokinet Biopharm 15: 241–253. 3668802 10.1007/BF01066320

[14] Friedman EB, Sun Y, Moore JT, Hung HT, Meng QC, et al (2010) A conserved behavioral state barrier impedes transitions between anesthetic-induced unconsciousness and wakefulness: evidence for neural inertia. PLoS One 5: e11903. 20689589 10.1371/journal.pone.0011903 PMC2912772

[15] Schwender D, Daunderer M, Mulzer S, Klasing S, Finsterer U, et al (1996) Spectral edge frequency of the electroencephalogram to monitor “depth” of anaesthesia with isoflurane or propofol. Br J Anaesth 77: 179–184. 8881621 10.1093/bja/77.2.179

[16] Billard V, Gambus PL, Chamoun N, Stanski DR, Shafer SL (1997) A comparison of spectral edge, delta power, and bispectral index as EEG measures of alfentanil, propofol, and midazolam drug effect. Clin Pharmacol Ther 61: 45–58. 9024173 10.1016/S0009-9236(97)90181-8

[17] Sleigh J, Steyn-Ross M, Steyn-Ross A, Voss L, Wilson M (2010) Anesthesia-Induced State Transitions in Neuronal Populations. In: Hudetz A, Pearce R, editors. Suppressing the Mind: Anesthetic Modulation of Memory and Consciousness. New York: Humana Press. 139–160.

[18] Gandomani MJ, Ghashghaii A, Tamadon A, Attaran HR, Behzadi MA, et al (2011) Comparison of Anaesthetic Effects of Ketamine -Xylazine and Ketamine- Diazepam Combination in Budgerigar. Vet Scan 6: 81.

[19] Fosse RT, Grong K, Stangeland L, Lekven J (1987) Anesthetic interaction in cardiovascular research models: effects of xylazine and pentobarbital in cats. Am J Vet Res 48: 211–218. 3826859

[20] Alkire MT (2010) Anesthesia and the Thalamocortical System. In: Hudetz A, Pearce R, editors. Suppressing the Mind: Anesthetic Modulation of Memory and Consciousness. New York: Humana Press. 127–138.

[21] Hudetz AG (2006) Suppressing consciousness: Mechanisms of general anesthesia. Seminars in Anesthesia, Perioperative Medicine and Pain 25: 196–204.

[22] Massimini M, Ferrarelli F, Huber R, Esser SK, Singh H, et al (2005) Breakdown of cortical effective connectivity during sleep. Science 309: 2228–2232. 16195466 10.1126/science.1117256

