# Supplementary figures and images for: Characterization of Phase Transition in the Thalamocortical System during Anesthesia-Induced Loss of Consciousness
Source: PLoS One. 2012 Dec 7;7(12):e50580. doi: 10.1371/journal.pone.0050580 (PMC3517525; doi:10.1371/journal.pone.0050580)

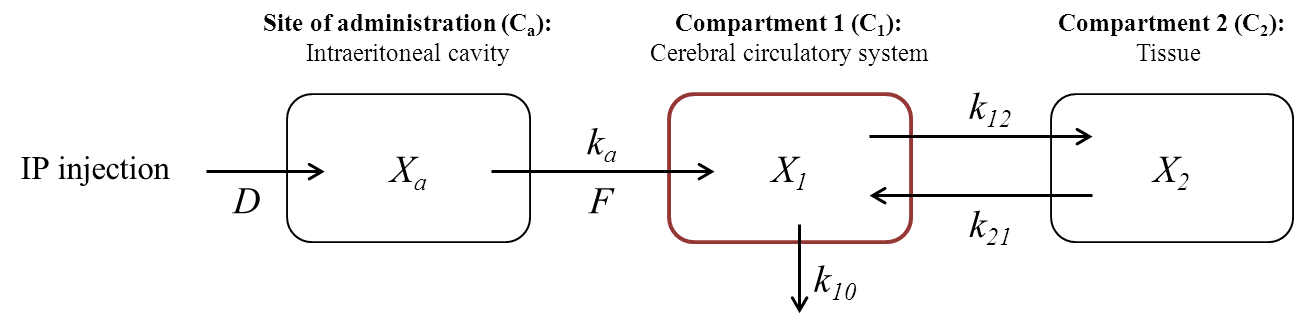

Supplement: Figure S1 — Diagram of three-compartment model. Xi denotes the amount of ketamine in the compartment Ci. The transition rates between compartments (kij) and the elimination rates from the site of drug administration (ka) and from the cerebral circulatory system (k10) are assumed to be first order. D and F mean dose of ketamine and bioavailability, respectively. The ketamine concentration at Ci is defined as the amount of ketamine at the compartment divided by the volume of the compartment. (TIF) [file pone.0050580.s001.tif]

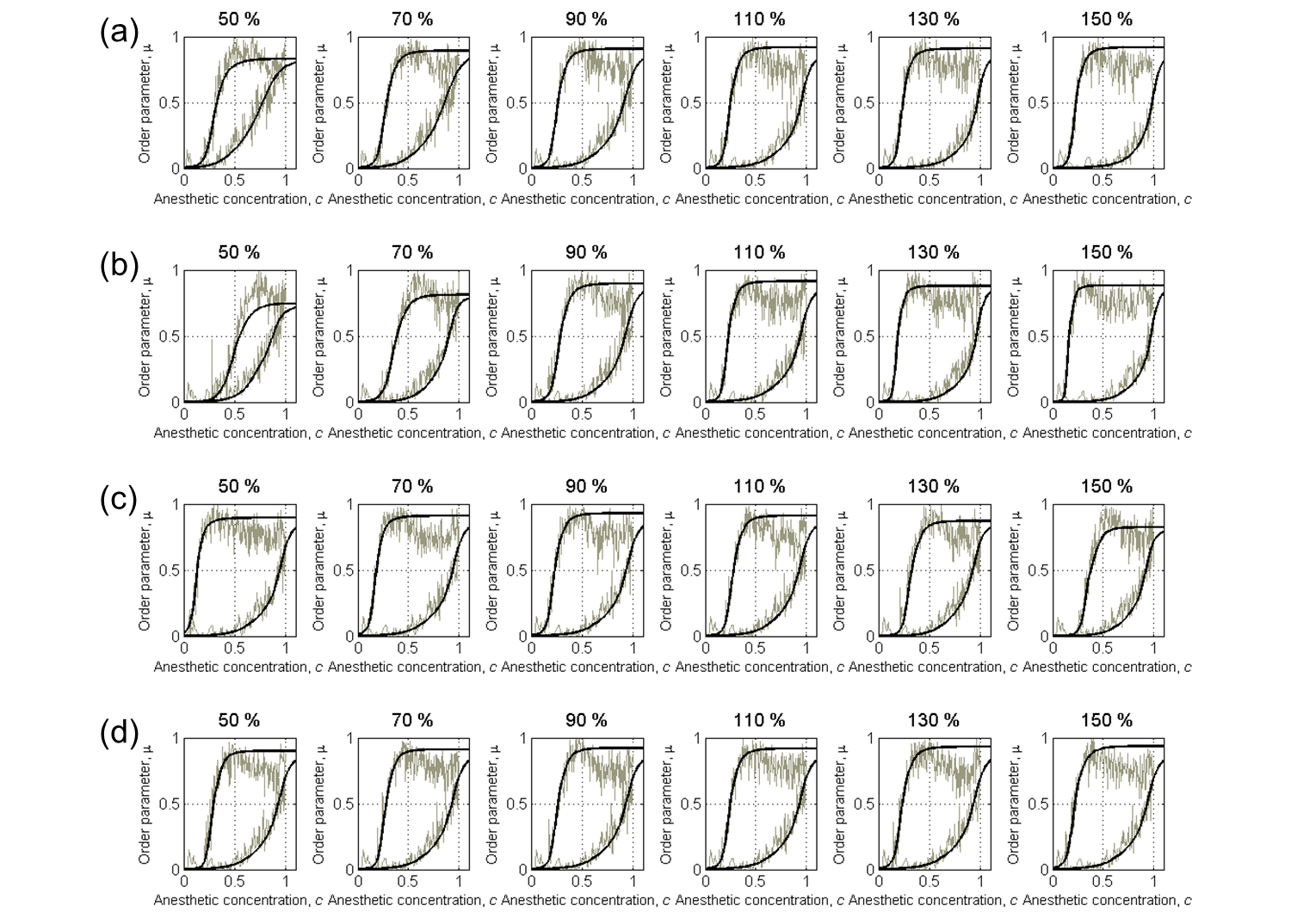

Supplement: Figure S2 — (a) Variation of order parameter curve with respect to varying ka . Order parameter curves are drawn with the anesthetic concentration profiles calculated with 50, 70, 90, 110, 130 and 150% values of estimated ka, from left to right. (b) Variation of order parameter curve with respect to varying α. Order parameter curves are drawn with the anesthetic concentration profiles calculated with 50, 70, 90, 110, 130 and 150% values of estimated α, from left to right. (c) Variation of order parameter curve with respect to varying k12. Order parameter curves are drawn with the anesthetic concentration profiles calculated with 50, 70, 90, 110, 130 and 150% values of estimated k12, from left to right. (d) Variation of order parameter curve with respect to varying β. Order parameter curves are drawn with the anesthetic concentration profiles calculated with 50, 70, 90, 110, 130 and 150% values of estimated β, from left to right. (TIF) [file pone.0050580.s002.tif]
