# Supplementary material for: Characterization of Phase Transition in the Thalamocortical System during Anesthesia-Induced Loss of Consciousness
Source: PLoS One. 2012 Dec 7;7(12):e50580. doi: 10.1371/journal.pone.0050580 (PMC3517525; doi:10.1371/journal.pone.0050580)
Supplement: Table S1 — The parameters without asterisks are fixed ones, determined from the plasma concentration-time profiles of ketamine. Those with asterisks are adjustable ones, derived from the time profile of plasma levels of ketamine and/or the behavioral reference points i.e., t ADM, t LOM, and t ROM. The values with dagger (†) were expressed as means±standard deviation. (DOCX) [file pone.0050580.s003.docx]

Table S1. Model Parameters

| **Parameter** | **Value** | **Unit** |
| --- | --- | --- |
| $k_{a}$ | *0.705 ± 0.265^†^ | min^-1^ |
| α | *0.111 ± 0.0246^†^ | min^-1^ |
| β | 0.00508 | min^-1^ |
| $k_{12}$ | 0.0684 | min^-1^ |
| $k_{21}$ | 0.0262 | min^-1^ |
| $k_{10}$ | 0.0215 | min^-1^ |
