# Supplementary material for: Characterization of Phase Transition in the Thalamocortical System during Anesthesia-Induced Loss of Consciousness
Source: PLoS One. 2012 Dec 7;7(12):e50580. doi: 10.1371/journal.pone.0050580 (PMC3517525; doi:10.1371/journal.pone.0050580)
Supplement: Information S1 — (DOCX) [file pone.0050580.s005.docx]

***Three-compartment model for ketamine concentration in brain tissue***

The three-compartment model is used to predict the concentration of ketamine in the brain. The model system is divided into three compartments: the first one (C_1_) encompasses the initial dilution volume, which is composed of the rapidly perfused tissues and organs. In considering the administered ketamine readily crosses the blood-brain-barrier and is rapidly distributed into the brain tissues ([White et al., 1982](#_ENREF_3)), we assumed that the ketamine concentration in the compartment-1 C_1_ is roughly proportional to the effective concentration needed for blocking the ketamine receptors such as NMDA receptor ([Schuttler et al., 1987](#_ENREF_2)). In addition, the elimination of ketamine is assumed as occurring directly and exclusively from C_1_. The second compartment (C_2_) involves the tissues and organs of the body which are less well perfused. Finally, an additional compartment (C_a_) which denotes site of administration of ketamine is incorporated into the model.

At *t=t_0_*, a quantity D of ketamine is administered into the site of administration denoted as compartment-a (C_a_). A fraction of F of the quantity D is then absorbed to the cerebral circulatory system (compartment-1, C_1_) from which it is both exchanged with compartment-2 (C_2_) and eliminated. This model assumes that all transfer processes follow first-order kinetics, and the brain is part of the C_1_.

Assuming the absorption of ketamine is first-order process, the dynamic equation for the amount of ketamine *X_a_* in C_a_ reads

$\frac{dX_{a}}{dt}={-k}_{a}X_{a}$ (S1.1)

where $k_{a}$ denotes first-order absorption rate constant after the ip administration. We next consider variations of the amount of ketamine in the C_1_ with time, which are determined by the difference between the influx and efflux rates of the ketamine. Denoting the amount of ketamine in the C_1_ by *X_1_*, we thus write the equation for the dynamics of the *X_1_*:

$\frac{dX_{1}}{dt}=k_{a}X_{a}-(k_{12}+k_{10}) X_{1}+k_{21}X_{2}$*,* (S1.2)

where $k_{12}$ and $k_{21}$ are the first-order transfer rate constant from C_1_ to C_2_ and C_2_ to C_1_, respectively. $k_{10}$ means the first-order elimination rate constant from C_1_. Note that in this model we assumed that ketamine elimination occurs only from the C_1_. The amount of ketamine in compartment-2 $X_{2}$ is given by

$\frac{dX_{2}}{dt}=k_{12}X_{1}-k_{21}X_{2}$. (S1.3)

Solving the equations with initial conditions $X_{1}\left( t_{0} \right)=X_{2}\left( t_{0} \right)=0$ and $X_{a}\left( t_{0} \right)=FD$ is straightforward to give the concentration of ketamine in each compartment at time *t* in the form:

$c_{a}\left( t \right)=\frac{FD}{V_{a}}e^{{-k}_{a}(t-t_{0})}$*,* (S1.4)

$c_{1}\left( t \right)=\frac{k_{a}FD(k_{21}-k_{a})}{V_{1}\left( \alpha-k_{a} \right)(\beta- k_{a})}e^{{-k}_{a}(t-t_{0})} + \frac{k_{a}FD(k_{21}-\alpha)}{V_{1}\left( k_{a}- \alpha\right)(\beta- \alpha)}e^{-\alpha(t-t_{0})}+ \frac{k_{a}FD(k_{21}-\beta)}{V_{1}\left( k_{a}- \beta\right)(\alpha- \beta)}e^{-\beta(t-t_{0})}$*,*

(S1.5)

$c_{2}\left( t \right)=\frac{k_{12}k_{a}FD}{V_{2}\left( \alpha-k_{a} \right)\left( \beta- k_{a} \right)}e^{{-k}_{a}\left( t-t_{0} \right)} + \frac{k_{12}k_{a}FD}{V_{2}\left( k_{a}- \alpha\right)\left( \beta- \alpha\right)}e^{-\alpha\left( t-t_{0} \right)}+ \frac{k_{12}k_{a}FD}{V_{2}\left( k_{a}- \beta\right)\left( \alpha- \beta\right)}e^{-\beta\left( t-t_{0} \right)}$

(S1.6)

With $\alpha=\left[ k_{10}+k_{12}+k_{21}+\sqrt{({k_{10}+k_{12}+k_{21})}^{2}-4k_{21}k_{10}} \right]/2$ and $\beta=\left[ k_{10}+k_{12}+k_{21}-\sqrt{({k_{10}+k_{12}+k_{21})}^{2}-4k_{21}k_{10}} \right]/2$, where $t_{0}$ denotes the administration time. Here, we set $t_{0}=0.$

With an assumption of linear relationship between $X_{1}$ and behavioral effect of ketamine, it is assumed that $k_{a}$ is inversely proportional to the interval between *t*_LOM_ and *t*_ADM_ with proportionality constant 1. The decay rate *α* is also assumed to be inversely proportional to the interval between *t*_ROM_ and *t*_LOM_ with an appropriate proportionality constant *c*, with which the mean value of *α*’s equals the first-order rate constant for distribution process calculated by conventional methods from the plasma concentration-time profile of ketamine in rodent ([Leung and Baillie, 1989](#_ENREF_1)). The other decay rate *β* and the rate constants $k_{12}$, $k_{21}$, and $k_{10}$ are also determined from the time-concentration profile of ketamine ([Leung and Baillie, 1989](#_ENREF_1)). The resulting parameter values are displayed in Table S1.

**Reference**

Leung LY, Baillie TA (1989) Studies on the biotransformation of ketamine. II--Quantitative significance of the N-demethylation pathway in rats in vivo determined by a novel stable isotope technique. Biomedical & environmental mass spectrometry 18:401-404.

Schuttler J, Stanski DR, White PF, Trevor AJ, Horai Y, Verotta D, Sheiner LB (1987) Pharmacodynamic modeling of the EEG effects of ketamine and its enantiomers in man. Journal of pharmacokinetics and biopharmaceutics 15:241-253.

White PF, Way WL, Trevor AJ (1982) Ketamine--its pharmacology and therapeutic uses. Anesthesiology 56:119-136.
