# Supplementary material for: Characterization of Phase Transition in the Thalamocortical System during Anesthesia-Induced Loss of Consciousness
Source: PLoS One. 2012 Dec 7;7(12):e50580. doi: 10.1371/journal.pone.0050580 (PMC3517525; doi:10.1371/journal.pone.0050580)
Supplement: Information S2 — (DOCX) [file pone.0050580.s006.docx]

***Robustness of the concentration model with regard to varying parameters***

To verify the robustness of the model, its dependence on parameter selection was examined with varying rate constants. The ensemble averaged order parameter curves were drawn with respect to the anesthetic concentration calculated while varying single rate constant and fixing the others. Variation of rate constants by ±50 % was tested and resultant variation of order parameters were shown in Figures.

Variation of *k_a_* up to ±50 % of the estimated parameter did not make qualitative changes in the order parameter curve with regard to anesthetic concentration (Fig. S2(a)). Variation of α resulted in wider range of variability in the order parameter curve, but the path-dependent nature was conserved even with ±50 % variation of α value (Fig. S2(b)). When α decreased by less than 30 % of estimated value or *k_a_* decreased by less than 20 % of estimated value, hysteresis curve collapsed. Variation of the parameters *k_12_* and β did not change the shape of order parameter curve significantly (Fig. S2(c-d)).
